# The Cbr-DPY-10(Arg92Cys) modification is a reliable co-conversion marker for CRISPR/Cas9 genome editing in *Caenorhabditis briggsae*

**DOI:** 10.17912/micropub.biology.000554

**Published:** 2022-04-20

**Authors:** Itai Antoine Toker, Oliver Hobert

**Affiliations:** 1 Columbia University, Department of Biological Sciences.; 2 Howard Hughes Medical Institute

## Abstract

The maturation of genome editing techniques dramatically broadens the range of organisms amenable to mechanistic investigation. *Caenorhabditis briggsae* is a nematode species related to *C. elegans* and a favored target for comparative studies. Here, we expand the repertoire of co-conversion markers to facilitate the screening and isolation of CRISPR/Cas9-edited lines in *C. briggsae* . Similar to its homologous *C. elegans* mutation, *Cbr-dpy-10(Arg92Cys)* is phenotypically easy to detect in its heterozygous form and is distinguishable from other combinations of *Cbr-dpy-* 10 alleles, a valuable feature for the reliable isolation of marker-free CRISPR/Cas9-edited animals.

**Figure 1. Generation and characterization of the dominant Cbr-dpy-10(ot1210[Arg92Cys]) allele.**
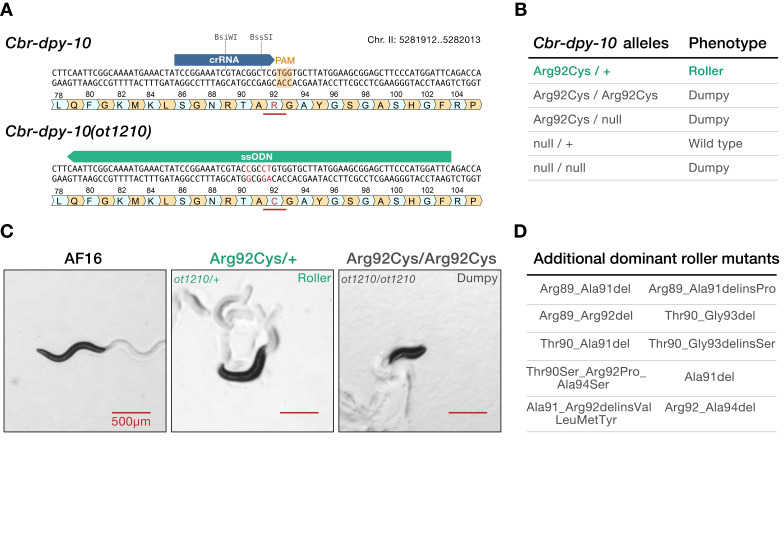
( **A** ) *Cbr-dpy-10* coding region near position Arg92, before (top) and after (bottom) precise genome editing using the CRISPR/Cas9 system with the indicated reagents. We used AF16 as a reference strain. Modified nucleotides and amino acids in *Cbr-dpy-10(ot1210)* are highlighted in red. The mutations disrupt restriction sites for the enzymes BsiWI and BssSI. ( **B** ) Phenotypes witnessed in animals bearing the indicated combinations of *Cbr* - *dpy-10* alleles. ( **C** ) Representative images of *C. briggsae* day-­1 adults. Genotypes appear above the images. Scale bar: 500μm. ( **D** ) Additional mutations we found to cause a dominant Roller phenotype in *C. briggsae* . Nomenclature according to HGVS recommendations (http://varnomen.hgvs.org/).

## Description

*Caenorhabditis briggsae* is a nematode species related to *C. elegans* that has become a prominent model for comparative biology. However, transgenesis and genome engineering protocols developed in *C. elegans* are consistently less efficient when applied to *C. briggsae* (Baird and Chamberlin 2006; Frøkjær-Jensen et al. 2014; Farboud et al. 2019). One proven means to increase the efficiency in obtaining CRISPR-Cas9-edited lines is to use a co-conversion strategy, whereby a second locus is targeted to generate an easily detectable phenotype used for selection and enrichment of potential successful recombinants at the locus of interest (Arribere et al. 2014; Paix et al. 2015).

We present and share reagent information for a co-conversion marker that facilitates the screening of CRISPR/Cas9-edited *C. briggsae* nematodes. In this approach, a point mutation generates an Arg to Cys conversion at position 92 of the Cbr-DPY-10 protein coding sequence ( **Figure 1A** ). We named the resulting allele *Cbr-dpy-10(ot1210)* . This mutation is homologous to a previously characterized and widely used co-conversion marker developed for *C. elegans* (Arribere et al. 2014; Paix et al. 2015). In both species, the mutation is dominant and generates an easily selectable “left-handed Roller” phenotype.

In *C. elegans* , the selectable mutation presents the particularity that the desired Arg92Cys/+ edits are phenotypically distinct from other allelic combinations (Arg92Cys/Arg92Cys, +/+, +/null, Arg92Cys/null, null/null) that could characterize the *dpy-10* loci upon CRISPR/Cas9 editing (Levy et al. 1993; Arribere et al. 2014). Our analyses determined this is also true for the homologous Arg92Cys mutation in *Cbr-dpy-10(ot1210)* ( **Figure 1B-C** ), granting it an advantage not found in co-conversion markers previously described for *C. briggsae* (Farboud et al. 2019; Cohen and Sternberg 2019). The phenotypic distinction between the different allelic configurations has several practical implications. The selection of F1 Rollers (Arg92Cys/+) ensures in advance that the picked individuals are heterozygous for the *Cbr-dpy-10(ot1210)* mutation and segregate unmarked (phenotypically wild-type) F2 individuals. Moreover, selection of F1 Rollers (Arg92Cys/+) and their distinction from +/null and Arg92Cys/null animals ensures that phenotypically wild-type F2 animals and their descendants do not carry additional mutations that could prevent reusing the *Cbr-dpy-10* locus for additional rounds of CRISPR/Cas9 editing with the same co-conversion reagents (Arribere et al. 2014). Alternatively, one can knowingly choose to select also F1 Dumpy worms (Arg92Cys/Arg92Cys, null/null or Arg92Cys/null) alongside F1 Rollers, to further increase the chances of detecting recombinants successfully edited at the unselected locus of interest. In that case, the investigator would anticipate that restoring the wild-type *Cbr-dpy-10* genetic background requires outcrossing and validation through sequencing.

To characterize null mutations resulting from CRISPR/Cas9 edits with the *Cbr-dpy-10* crRNA, we injected CRISPR mixes without a DNA repair template and randomly selected phenotypically wild-type F1s for further cultivation. 55% (22/40) of those F1s had ~25% of Dumpy F2 progeny, selected Dumpy worms displayed 100% Dumpy progeny, and Sanger sequencing of Dumpy animals revealed they are homozygous to frameshift indels. These results strongly suggest that nonsense mutations occurring near the Arg92 position are recessive and generate a Dumpy phenotype, similarly to homologous mutations in *C. elegans* (Arribere et al. 2014). Surprisingly, the no-repair *Cbr-dpy-10* CRISPR/Cas9 experiment also generated Rollers in the F1 descendants of all 10 injected P0 worms. Analysis of 14 Roller-derived lineages showed that the progeny of F1 Rollers phenotypically segregated similarly to the progeny of Arg92Cys/+ animals (~25% wild type, ~50% Roller, ~25% Dumpy), suggesting dominant mutations. Sequencing analyses revealed that in all 14 lineages, the causal mutations were small in-frame deletions or other in-frame mutations near the Arg92 position ( **Figure 1D** ). These results imply that a wide variety of in-frame mutations modifying the Cbr-DPY-10 protein near position Arg92 act as dominant Roller mutations. Whether this is true also to homologous mutations in *C. elegans dpy-10* remains to be determined.

Two co-conversion markers were previously described for *C. briggsae* (Farboud et al. 2019; Cohen and Sternberg 2019). In both cases, the dominant selectable mutation displays the same phenotypic outcome in heterozygotes and homozygotes, and therefore does not enjoy advantages stemming from a phenotypic distinction. Farboud et al. characterized the introduction of an early nonsense mutation in *Cbr-ben-1* that confers resistance to benzimidazole-induced paralysis. This approach is slightly more burdensome as it requires the preparation of benzimidazole-containing plates, but it is a very useful complement to the *Cbr-dpy-10(ot1210)* approach in cases where the non-selectable locus of interest is on chromosome II. Indeed, *Cbr-dpy-10* and *Cbr-ben-1* are respectively located on chromosomes II and III. Cohen and Sternberg reported that the insertion of a universal STOP-IN cassette at position Tyr24 of the *Cbr-dpy-10* sequence caused a dominant Dumpy phenotype (Cohen and Sternberg 2019). The reason for the difference in the phenotypic outcomes of nonsense alleles near the Arg92 position vs. the Tyr24 position is unclear. It could be due to partial and/or modified activity of short Cbr-DPY-10 protein products, to a combined effect on *Cbr-dpy-10* and a second gene *CBG31161* whose coding regions are both disrupted upon insertion of the STOP-IN cassette, or to another unknown reason.

## Methods

*C. briggsae* worms were cultivated at 23ºC on NGM plates supplemented with OP50 *E. coli* .

Cas9 Ribonucleoprotein mixtures were prepared as described in (Dokshin et al. 2018). The Cas9 protein (250ng), tracrRNA (2μg) and crRNA (1.12μg) were first mixed together and incubated at 37ºC for 15 minutes. Next, the ssODN donor (2.2μg) was added and the mixture brought to a final volume of 20μl using nuclease-free water.

For imaging of *C. briggsae* day-1 adults, L4 worms were transferred into fresh OP50-coated plates and grown for 20 hours at 23ºC. Images were acquired at room temperature using the WormLab automated multi-worm tracking system (MBF Bio-science).

## Reagents

***Cbr-dpy-10 crRNA* (5’- 3’)** (IDT, stock solution 0.4μg/μl) **:**

UCCGGAAAUCGUACGGCUCG

***Cbr-dpy-10(Arg92Cys)* ssODN (5’- 3’) (** IDT, stock solution 1μg/μl) **:**

GAATCCATGGGAAGCTCCGCTTCCATAAGCACCACAGGCGGTACGATTTCCGGATAGTTTCATTTTGCCGAATTG

Recombinant S. pyogenes Cas9 Nuclease V3 (IDT #1081059, stock solution 10μg/μl).

tracrRNA (IDT #1072532, stock solution 0.4μg/μl).

*C. briggsae* strains:

**Table:** 

Strain	Genotype	Available From
AF16	Reference strain	CGC
OH17874	*Cbr-dpy-10(ot1210) II*	CGC
