# Corrigendum: The Cbr-DPY-10(Arg92Cys) modification is a reliable co-conversion marker for CRISPR/Cas9 genome editing in *Caenorhabditis briggsae*

**DOI:** 10.17912/micropub.biology.001142

**Published:** 2024-02-02

## Description

For Toker, IA; Hobert, O (2022). The Cbr-DPY-10(Arg92Cys) modification is a reliable co-conversion marker for CRISPR/Cas9 genome editing in Caenorhabditis briggsae. microPublication Biology. 10.17912/micropub.biology.000554.

  In the version of the microPublication originally published online, the quantity of Cas9 protein was incorrectly stated as 250 ng and should be 5 μg instead. This mistake has been corrected online.

Original:

Cas9 Ribonucleoprotein mixtures were prepared as described in (Dokshin et al. 2018). The Cas9 protein (250ng), tracrRNA (2μg) and crRNA (1.12μg) were first mixed together and incubated at 37ºC for 15 minutes. Next, the ssODN donor (2.2μg) was added and the mixture brought to a final volume of 20μl using nuclease-free water.

Corrected to:&nbsp;

Cas9 Ribonucleoprotein mixtures were prepared as described in (Dokshin et al. 2018). The Cas9 protein (5μg), tracrRNA (2μg) and crRNA (1.12μg) were first mixed together and incubated at 37ºC for 15 minutes. Next, the ssODN donor (2.2μg) was added and the mixture brought to a final volume of 20μl using nuclease-free water.

